# Spontaneous Right-Sided Diaphragmatic Hernia: A Rare Cause of Small Bowel Obstruction

**DOI:** 10.7759/cureus.59279

**Published:** 2024-04-29

**Authors:** Phoebe Douzenis, Ali Yasen Y Mohamedahmed, Sreekanth Sukumaran, Zbigniew Muras, Najam Husain

**Affiliations:** 1 General Surgery, Queen's Hospital Burton, University Hospitals of Derby and Burton NHS Trust, Burton on Trent, GBR; 2 General Surgery, The Royal Wolverhampton NHS Trust, Wolverhampton, GBR; 3 Colorectal Surgery and General Surgery, University Hospitals of Derby and Burton, Burton on Trent, GBR

**Keywords:** hernia repair, small bowel resection, urgent laparotomy, small intestinal obstruction, diaphragmatic hernia

## Abstract

Diaphragmatic hernia (DH) is an uncommon cause of small bowel obstruction (SBO), particularly in the absence of trauma. This rarity can pose a diagnostic challenge, leading to significant delays in treatment and increased morbidity. We report a case of a 79-year-old male patient who presented with acute signs of small bowel obstruction. The patient had no reported history of trauma. Computed tomography (CT) of the abdomen revealed a diaphragmatic hernia causing small bowel obstruction. The patient underwent an initial laparoscopy, which was converted to laparotomy, small bowel resection, and subsequent hernia repair. The patient made a good recovery, and two weeks after his initial presentation, he was discharged home. This case highlights the importance of considering diaphragmatic hernia in differential diagnosis for small bowel obstruction, even in the absence of trauma.

## Introduction

A diaphragmatic hernia (DH) occurs when abdominal contents protrude into the thoracic cavity due to a defect within the diaphragm [1]. DH can be congenital or acquired. Congenital diaphragmatic hernia (CDH) is the most common type and refers to a developmental defect of the diaphragm. It typically presents in newborns with respiratory distress in the first few hours of life. The incidence of CDH varies significantly across the population and is estimated to be between 0.8 and 5/10,000 births. It is slightly more common in males than in females [2]. Left-sided CDH is more common than right-sided CDH and accounts for about 75% of cases. However, right-sided CDH is often associated with higher morbidity and mortality [3]. CDH can be classified into two types: Morgagni hernia and Bochdalek hernia. Bochdalek hernias are more common and present as a defect in the left posterolateral diaphragm, while Morgagni hernias present as an anterior defect [4].

Acquired diaphragmatic hernias (ADH) occur most often secondary to blunt or penetrating trauma to the abdomen, which results in diaphragmatic rupture [1]. However, ADH can also be iatrogenic following surgery. Diaphragmatic injuries are generally uncommon and represent less than 1% of all traumatic injuries [5]. Diaphragmatic rupture from trauma occurs in about 0.8%-3.6% of cases, with incidents of herniation following such injuries being relatively low [6,7]. The left side is more commonly affected than the right side in ADH. Injury to the left side of the hemidiaphragm is estimated to occur about three times more often than the right side [8].

We report a case of a right-sided anterior diaphragmatic hernia with no associated history of trauma.

## Case presentation

**Figure 1:**
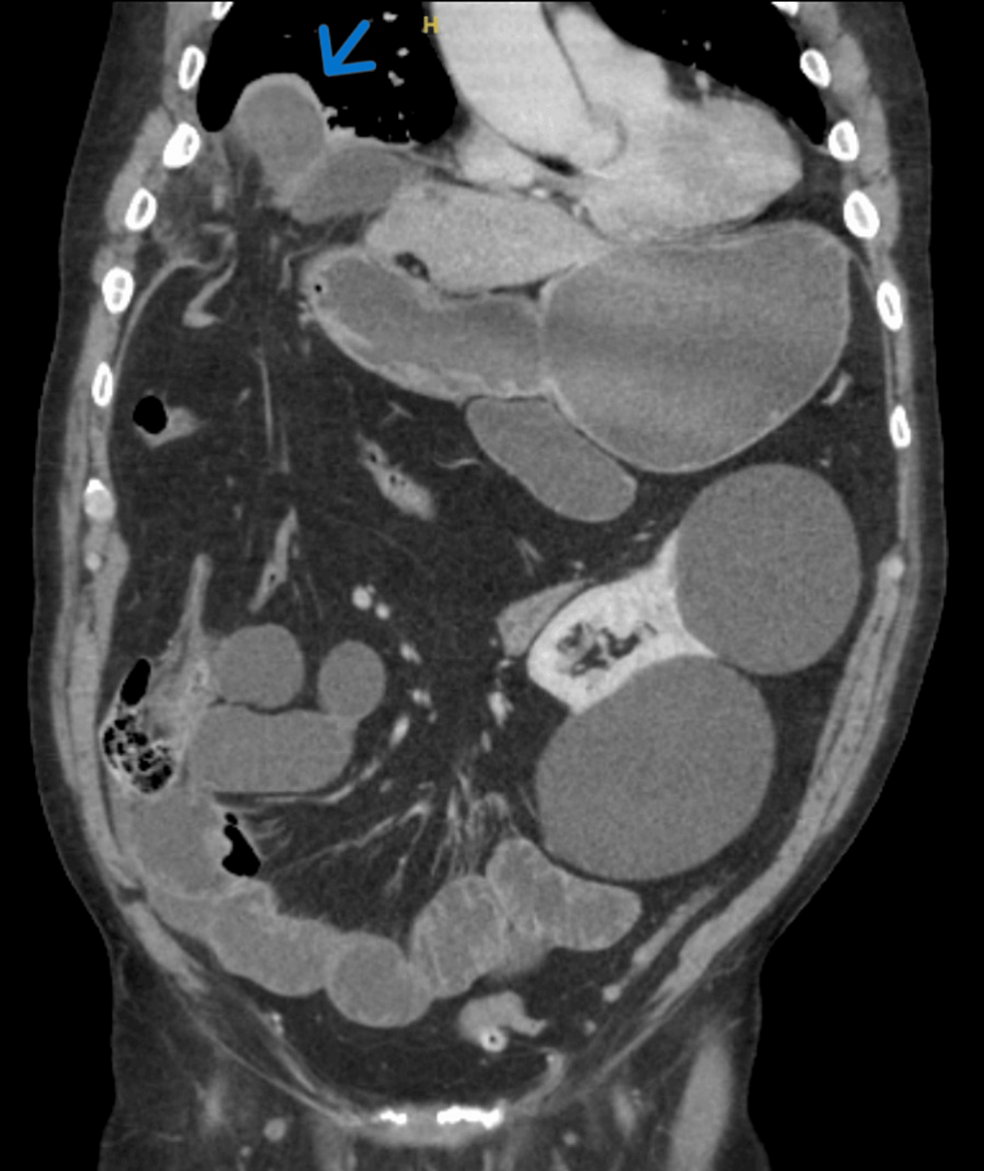
Abdominal and pelvic CT scan demonstrating small bowel obstruction and right-sided diaphragmatic hernia (blue arrow) CT: computed tomography

A 79-year-old male presented to Queen's Hospital Burton, United Kingdom, complaining of two days of increasing pain in the upper right quadrant of his abdomen, vomiting, and constipation. He had no history of trauma and had previously experienced gastric acid reflux and hypertension. On admission, he was hemodynamically stable, but there was tenderness and guarding in the upper abdomen. Blood tests revealed a raised lactate level of 4.7 mmol/L, a white blood cell count (WCC) of 14.7×10^9^/L, and a C-reactive protein (CRP) level of 187 mg/L. The possible diagnoses were a perforated peptic ulcer or acute cholecystitis. A computed tomography (CT) scan of his abdomen and pelvis revealed a right-sided anterior diaphragmatic hernia, causing a small bowel obstruction (SBO) (Figure 1).

**Figure 2:**
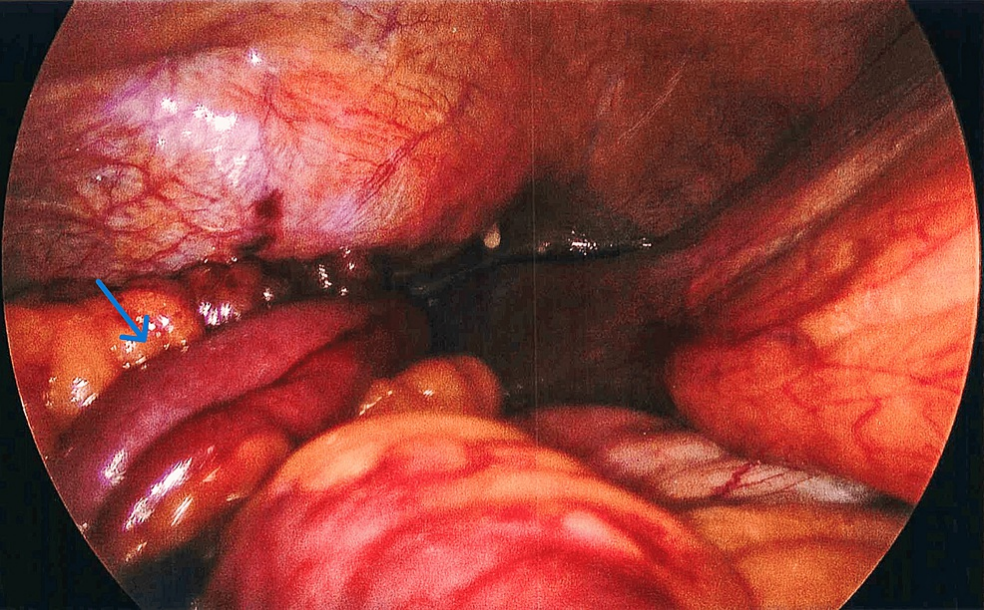
Intraoperative image demonstrating the small bowel loop (blue arrow) herniated through the diaphragmatic hernia

Following resuscitation, he underwent a laparoscopic converted to open repair of the strangulated diaphragmatic hernia and small bowel resection, as well as right-sided chest tube insertion. On laparoscopy, the diaphragmatic hernia on the right side in the anterior hemidiaphragm above the liver was visualized with a loop of small bowel entering the hernia defect with a dilated proximal loop and collapsed proximal loop (Figure 2).

**Figure 3:**
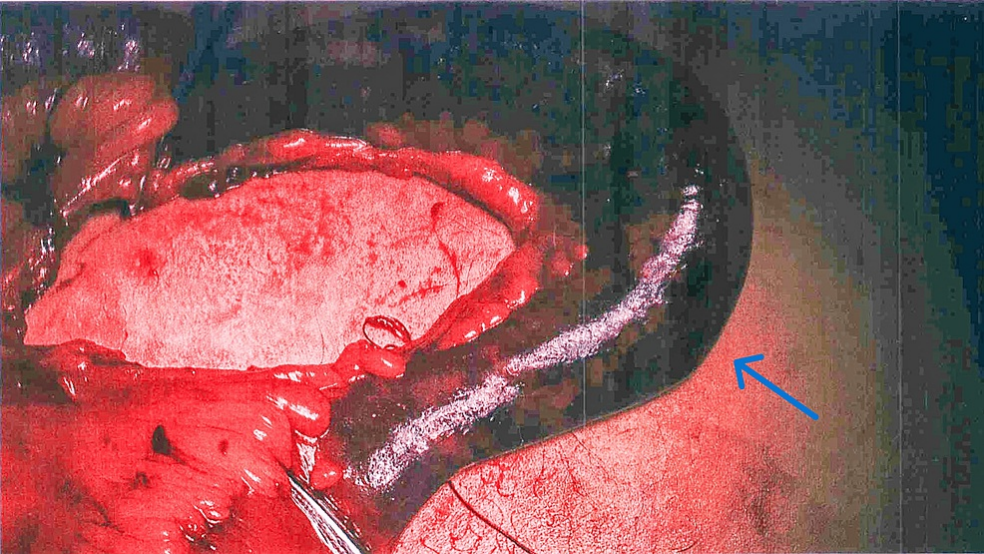
Intraoperative image demonstrating gangrenous small bowel loop (blue arrow) reduced from the hernia defect

The hernia defect contained a gangrenous small bowel loop, estimated to be around 15 cm long, with no bowel perforation (Figure 3).

There was no hernia sac; the 4 cm defect contained a bowel loop herniated directly in the right pleural cavity. The necrotic omentum and the gangrenous bowel were resected, and intestinal continuity was restored with a side-to-side small bowel anastomosis. A 28-French chest tube was inserted in the right-sided pleural cavity. Finally, the hernia defect in the diaphragm was repaired with two layers.

Postoperatively, he was transferred to the intensive care unit (ICU), extubated on the same day as his operation, and remained in the ICU for seven days. He developed right-sided pleural effusion, suggestive of infection, which was treated with antibiotics. The patient made a good recovery, and two weeks after his initial presentation, he was discharged home.

## Discussion

Small bowel obstruction accounts for 80% of all bowel obstructions, with a similar incidence seen in males and females but with a higher incidence with increasing age [9]. Small bowel obstruction (SBO) is estimated to occur in about 4.6% of patients after intra-abdominal surgery, with adhesions being the most common cause, accounting for up to 70% of cases of SBO [10]. However, in patients with no previous abdominal surgery or procedures, abdominal wall hernias are the most common cause of SBO [10]. Incarcerated hernias in the inguinal, femoral, ventral, or umbilical regions are usually identified on early abdominal examinations and are typically managed through urgent surgical interventions. Interestingly, a meta-analysis published in 2020 concluded that the most common cause of SBO in the virgin abdomen was malignancy [11].

SBO is a common indication for emergency laparotomy in the United Kingdom, with an estimated 90-day mortality of around 13%, with similar values seen in the United States of America [12]. Similar to the presentation of this reported case, SBO is often associated with vomiting, usually due to the proximal bowel distention; the twisting of the bowel can lead to reduced blood flow, which further results in edema and inflammation, which increases the risk of ischemia and perforation [9].

Diaphragmatic hernias, the cause for SBO in the reported case, are usually congenital or associated with a history of trauma [1,13]. There is limited literature and case reports of SBO associated with non-traumatic diaphragmatic hernias in adults [14]. Moreover, large bowel obstruction due to DH has been reported in the literature [15]. Diaphragmatic hernias are associated with a range of potential complications, including diaphragmatic rupture, acute obstructive symptoms, respiratory failure, incarceration and strangulation of the intestine, and cardiac tamponade [1,13,14].

Management of diaphragmatic hernia involves a multidisciplinary approach tailored to the individual patient's condition, considering factors such as the type of hernia (congenital or acquired), symptoms, and overall health status [2]. In cases of congenital diaphragmatic hernia (CDH), especially in newborns, surgical intervention is typically required shortly after birth to reposition the abdominal organs into the correct place and repair the diaphragm [1,16]. In situations where primary closure is not possible due to the size of the defect, mesh repairs can be used, and in some cases, laparoscopic approaches may be feasible [17]. Literature showed various approaches for incarcerating right-sided DH, such as emergency laparotomy [15,18] and minimally invasive repair through laparoscopy [19]. In this case report, laparoscopic repair was not feasible due to dilated bowel loops necessitating conversion to laparotomy to achieve a better view and access to the hernia site, encompassing the interventions reported in previous similar cases.

## Conclusions

Diaphragmatic hernias are a rare cause of bowel obstruction that is typically congenital and seen in younger patients. In adult patients, DH is often associated with trauma, and rarely, similar to this case, there is no history of trauma. This case report highlights the need for vigilance in similar cases to ensure early diagnosis and intervention to minimize the risk of further complications.
